# Squamous cell carcinoma of the vulva diagnosed by a dermatologist^☆^^☆☆^

**DOI:** 10.1016/j.abd.2019.04.005

**Published:** 2019-11-06

**Authors:** Isadora Barreto Michels, Cláudio Sampieri Tonello, Cleverson Teixeira Soares

**Affiliations:** a Department of Dermatology, Instituto Lauro de Souza Lima, Bauru, SP, Brazil; b Department of Pathology, Instituto Lauro de Souza Lima, Bauru, SP, Brazil

Dear Editor;

Vulvar cancer is considered a rare disease, accounting for approximately 4% of all female genital neoplasms, and squamous cell carcinoma is the most prevalent, accounting for 90% of malignant tumors in this region, followed by melanoma. Its incidence is higher in the elderly population, with a worldwide incidence of approximately 1.8/100,000 women, increasing to 20/100,000 after the age of 75 years.^1^, ^2^, ^3^, ^4^

A female patient, 82 years old, from Bauru-SP, was referred to the dermatology department with complaint of pruritus and a lesion in the genital region two months previously. Hypertension, hypothyroidism, and diabetes were reported as comorbidities. Widowed 14 years ago, she denied sexual activity ever since.

On examination of the genital region, erythema and edema of the entire labia majora were found, and an ulcerative nodule approximately 1.7 cm in diameter was found in the region of the labia minora (Fig. 1).

**Figure 1:**
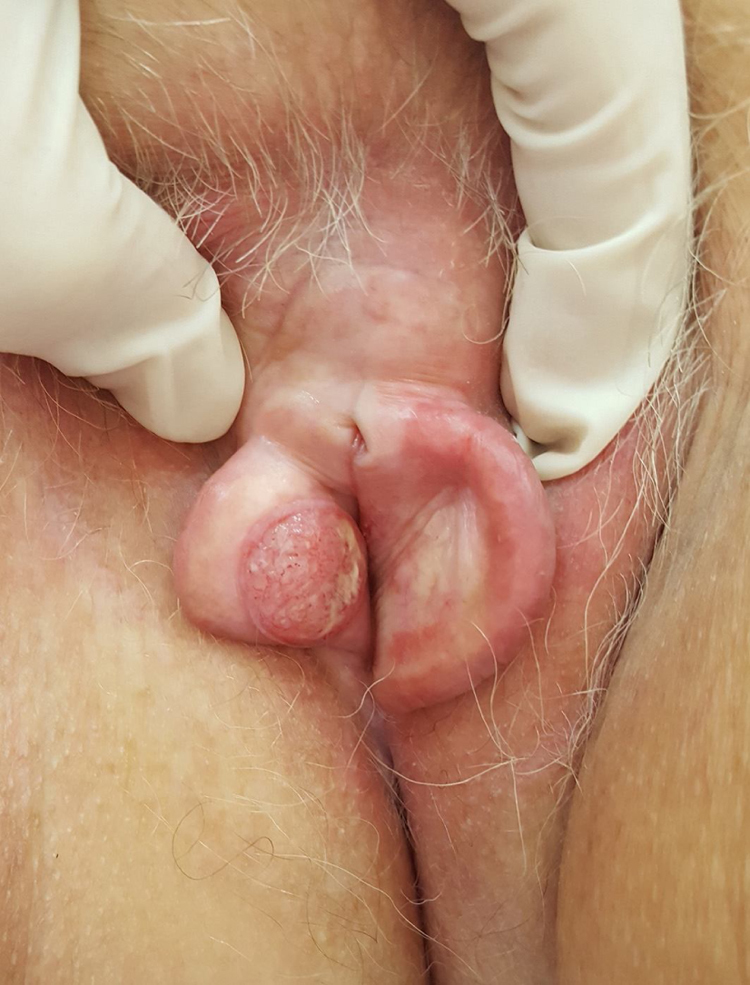
Ulcerated nodular tumor infiltrating the labia minora.

An incisional biopsy was performed, showing a well-differentiated, invasive, ulcerated squamous cell carcinoma (Fig. 2). HPV screening for genome amplification was negative (Table 1). The patient was referred to the oncology department for treatment at a referral hospital.

**Figure 2:**
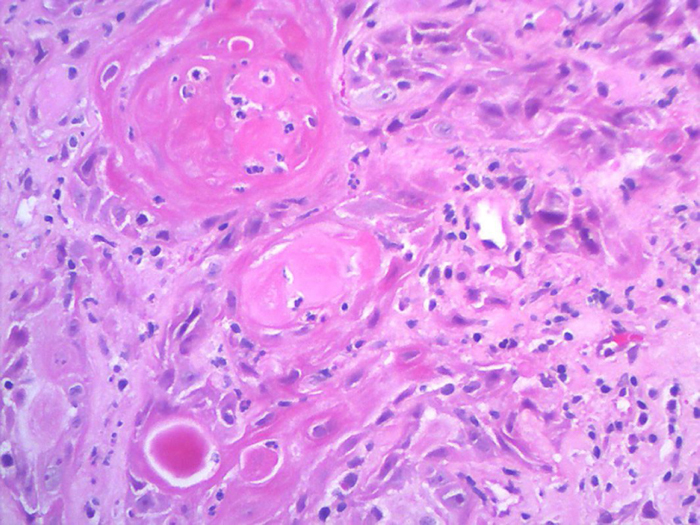
Blocks of atypical epithelial cells with corneal pearl formation (Hematoxylin & eosin, ×200).

**Table 1:** HPV screening by genome amplification.

Target sequences tested	Result
HPV SOE (184 pb)	Negative
HPV 6 (118 pb)	Negative
HPV 11 (120 pb)	Negative
HPV 16 (137 pb)	Negative
HPV 18 (121 pb)	Negative

Squamous cell carcinoma of the vulva usually presents as a solitary nodule or ulcer on the labia majora or minora, with associated pain, bleeding, itching, odor, or discharge. Among the causative factors involved in its pathogenesis are smoking, HPV infection, and precursor lesions such as lichen-sclerotic atrophy and incipient vulvar carcinomas – now called vulvar intraepithelial neoplasms – also related to HPV.^2^

Morphological variants have been described, including basaloid and verrucoid subtypes, which are related to viral infection and are more common in the young population, whereas keratinizing variants tend to be HPV-negative and occur in elderly women.^1^

The staging is performed by the TNM system. Tumors diagnosed early, up to stage T1a, can be treated only with resection of the lesion with at least 1 cm of margin, with survival rates at five and ten years around 100% and 94.7%, respectively. From the T1b stage, tumors from 2 cm with stromal invasion of at least 1 mm may require partial or total vulvectomy, with ipsilateral or bilateral lymph node dissection. Since lymphatic dissemination is the most important prognostic factor, histopathological study of regional lymph nodes is essential in cases of invasive tumors. If lymph node invasion is confirmed, chemotherapy and additional radiation therapy may be required.^3^, ^5^

Treatment in the early stages has better results, both esthetic-functional and in terms of overall survival, presenting a high cure rate; however, it occurs in a very low percentage of women affected, since in general they seek medical services late and professionals delay in making the diagnosis.

The dermatological literature lacks of scientific articles of the neoplasms of the female genital epithelium; the present report attempts to demonstrate the importance of the anamnesis and the dermatological examination of this anatomical region in order to make an early diagnosis.

## Financial support

None declared.

## Authors’ contribution

Isadora Barreto Michels: Conception and planning of the study; composition of the manuscript; collection, analysis, and interpretation of data; critical review of the literature.

Cláudio Sampieri Tonello: Approval of the final version of the manuscript; participation in the design of the study; intellectual participation in the propaedeutic and/or therapeutic conduct of the studied cases; critical review of the manuscript.

Cleverson Teixeira Soares: Approval of the final version of the manuscript; collection, analysis, and interpretation of data.

## Conflicts of interest

None declared.
